# The Compatible Solute Ectoine Reduces the Exacerbating Effect of Environmental Model Particles on the Immune Response of the Airways

**DOI:** 10.1155/2014/708458

**Published:** 2014-04-13

**Authors:** Klaus Unfried, Matthias Kroker, Andrea Autengruber, Marijan Gotić, Ulrich Sydlik

**Affiliations:** ^1^IUF Leibniz Research Institute for Environmental Medicine, Auf'm Hennekamp 50, 40225 Düsseldorf, Germany; ^2^Department of Material Chemistry, Ruđer Bosković Institute, Bijenička cesta 54, 10000 Zagreb, Croatia

## Abstract

Exposure of humans to particulate air pollution has been correlated with the incidence and aggravation of allergic airway diseases. In predisposed individuals, inhalation of environmental particles can lead to an exacerbation of immune responses. Previous studies demonstrated a beneficial effect of the compatible solute ectoine on lung inflammation in rats exposed to carbon nanoparticles (CNP) as a model of environmental particle exposure. In the current study we investigated the effect of such a treatment on airway inflammation in a mouse allergy model. Ectoine in nonsensitized animals significantly reduced the neutrophilic lung inflammation after CNP exposure. This effect was accompanied by a reduction of inflammatory factors in the bronchoalveolar lavage. Reduced IL-6 levels in the serum also indicate the effects of ectoine on systemic inflammation. In sensitized animals, an aggravation of the immune response was observed when animals were exposed to CNP prior to antigen provocation. The coadministration of ectoine together with the particles significantly reduced this exacerbation. The data indicate the role of neutrophilic lung inflammation in the exacerbation of allergic airway responses. Moreover, the data suggest to use ectoine as a preventive treatment to avoid the exacerbation of allergic airway responses induced by environmental air pollution.

## 1. Introduction

The exposure of humans to particulate air pollution has been correlated with the incidence of atopic allergies [1]. In particular, traffic-related air pollution is strongly linked to allergic diseases including asthmatic bronchitis [2]. It is hypothesized that an adjuvant effect of inhaled particles may influence either the process of sensitization or the immune response, at the level of the disease outcome [3]. In asthma patients, such adverse effects of particulate air pollution can be observed as an acute exacerbation of allergic lung inflammation [4–6].

Current research is focusing on the molecular mechanisms by which such a toxic potential of environmental particles is mediated. As one common denominator of particle-induced adverse health effects, oxidative stress in the airways has been identified [7]. Reactive oxygen species may be triggered by the intrinsic oxidative potential of inhaled particulate matter which depends on chemical properties like elemental composition and surface charges. But also via indirect cell mediated pathways oxidative stress is generated in the airways. Upon cell contact, in particular ultrafine or nano-sized particles may interact with cellular components and organelles which can contribute to the production of reactive oxygen species [8]. Additionally, the induction of an inflammatory response, which is a typical reaction to the inhalation of poorly soluble material, can lead to an oxidative burst from inflammatory cells like macrophages and neutrophilic granulocytes [9].

So far, it is not clear whether all these potential mechanisms contribute to the exacerbation of immune reactions of the airways or whether one of these pathways dominates the adverse effects and might therefore be a useful target for preventive and therapeutic approaches. In the system of ovalbumin (OVA)-sensitized mice this problem was addressed by inhalation studies with pure carbon nanoparticles (CNP). Such particles are considered model particles for combustion-derived recent particulate air pollution. Inhalation of these particles prior to OVA challenge led to an aggravation of the allergic airway responses including infiltration of inflammatory cells and excretion of cytokines [10]. An intervention study employing antioxidants in this scenario demonstrated that a reduction of the oxidative stress prevents the exacerbation of the airway response [11]. Using this experimental system, it should be possible to test the preventive value of compounds for individuals who suffer from atopic asthma, which might be exacerbated after inhalation of particulate matter.

In our earlier studies, we were able to demonstrate that the compatible solute ectoine is able to reduce the neutrophilic inflammatory response of the airways after exposure to pure (CNP). In the system of particle-exposed rats, the neutrophilic lung inflammation was significantly reduced when ectoine was present during exposure [12]. At a mechanistic level, we were able to demonstrate that ectoine prevents the activation of proinflammatory reactions in lung epithelial cells by stabilizing membrane signalling platforms which are addressed by oxidative cell stress coming by the particles [13]. In this context, it has been shown that ectoine does not interact with the particles themselves. The stabilizing mechanism of ectoine was investigated by a number of “proof of principle” experiments, which demonstrated that the mechanism is not based on antioxidant properties of the substance. Additionally, investigating the effect of ectoine during an ongoing neutrophilic inflammation, we observed the prevention of antiapoptotic and therefore proinflammatory reactions of neutrophils in the inflammatory environment by ectoine [14]. This effect was observed not only in the animal system, where it led to an accelerated resolution of the inflammation, but also in the human system employing peripheral neutrophils from patients suffering from chronic obstructive pulmonary disease (COPD).

The possibility to reduce neutrophilic lung inflammation without directly interfering with the oxidative potential of the nanoparticles offers the possibility to investigate the relevance of the neutrophilic inflammation for the exacerbation of allergic lung inflammation. Ectoine is a highly compatible substance which is tolerated by higher organisms without any known side effects. Therefore, the system might give indications for possible therapeutic value for asthma patients. For that purpose, the experimental system of CNP-induced neutrophilic lung inflammation was adopted to C57/Bl6 mice and the effect of ectoine application was evaluated in these organisms. The influence of ectoine of local and systemic cytokines with immune relevance was tested. In a second step, the system was applied to OVA-sensitized mice which were challenged in the presence of CNP and ectoine.

## 2. Materials and Methods

### 2.1. Particle Suspensions

Carbon nanoparticles (CNP, Printex 90) were obtained from Degussa (Essen, Germany). Stock suspensions (1 mg/mL) of particles were prepared in phosphate buffered saline (PBS) by sonication for 60 min. Particles and particle suspensions were characterized by (i) scanning electron microscopy (JSM 7000F, JEOL Ltd., Japan), (ii) BET using FlowSorb II 2300 analyzer (Micromeritics, Norcross, USA), and (iii) light scattering using ZetratracTM NPA152 (Microtrac, Montgomeryville, PA, USA). Particle suspension characteristics were as described previously [13].

### 2.2. Animal Experiments

Female C57BL/6JRj mice (8 weeks old, Janvier, France) were treated with particle suspensions or control solutions via pharyngeal aspiration with a volume of 50 *μ*L, under inhalation anaesthesia (isoflurane, 5% in synthetic air, 1-2 min). Animals were sensitized by repetitive intraperitoneal injection of 1 *μ*g OVA/alum. At the indicated time point, mice were challenged by aerosol inhalation (1% OVA in PBS) for 30 min using a Pari-Boy Nebuliser (Pari, Starnberg, Germany). Animals were sacrificed by exsanguination under anaesthesia after the indicated exposure times. Serum was prepared from blood samples taken prior to exsanguination. Bronchoalveolar lavage was taken using 4 × 1 mL PBS. All animal experiments were performed after relevant permission according to German animal protection laws.

### 2.3. Lavage Parameters

Differential cell counts were performed from Giemsa/May-Grünwald stainings of lavage cells. Cell free lavage fluids were subjected either to solid-phase ELISA in order to determine KC and IL-6 (R&D systems, Minneapolis, MN) or to Mouse Cytokine Antibody Arrays (RayBiotech, Inc., Norcross, CA) according to the respective manufacturer's instructions. Signal strengths of the arrays were determined densitometrically from autoradiographs using Quantity One software (version 4.1, Biorad, Hercules, CA, USA). IL-6 serum content was determined using the above-mentioned ELISA system.

### 2.4. Statistical Analyses

**Figure 1:**
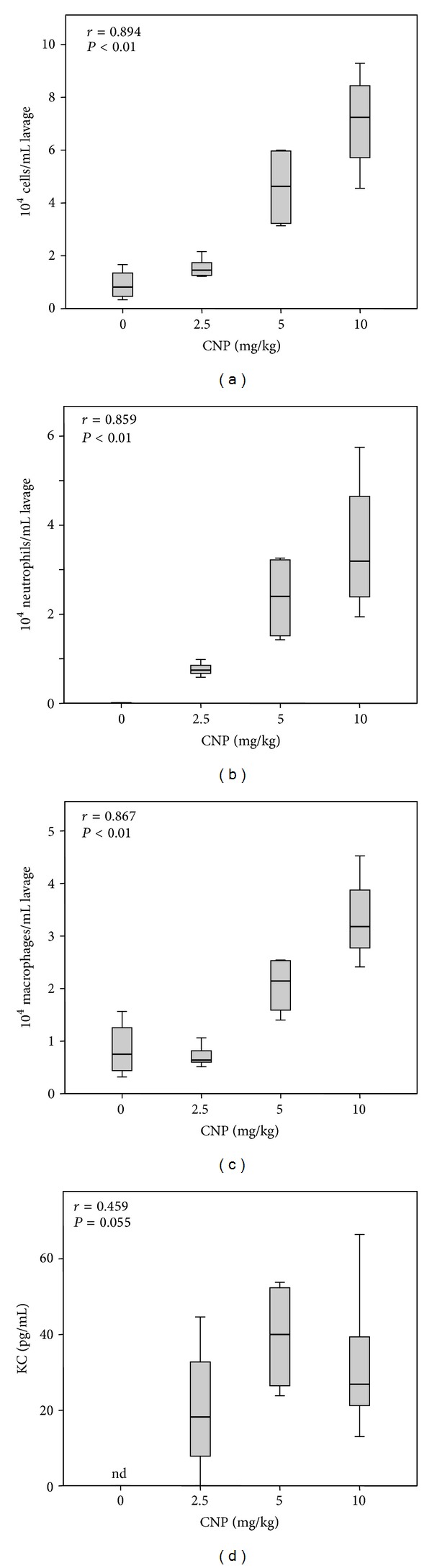
Lung inflammation induced by increasing doses of CNP. Female C57/Bl6 mice (8 weeks old) were exposed once to the indicated dose of CNP suspended in PBS. Animal numbers were 0 mg/kg *n* = 4, 2.5 mg/kg *n* = 5, 5 mg/kg *n* = 4, and 10 mg/kg *n* = 5. Inflammation parameters were determined 24 h after exposure. (a) Total number of cells per mL BAL; (b) total number of neutrophilic granulocytes per mL BAL; (c) total number of macrophages and monocytes per mL BAL; (d) pg/mL of KC in BAL. *r*, correlation coefficient of Pearson correlation; *P*, two sided significance; nd, not detectable.

Statistical calculations were performed using IBM SPSS statistics 22. Significant values were calculated either by ANOVA analyses with Tukey's HSD post hoc testing or by comparison of individual groups by Mann-Whitney *U*-test. Dose response relationships were analysed by Pearson correlations. Except for the boxplot in Figure 1, mean values with standard errors are given. Power calculations for the design of animal experiments were performed using G*Power version 3.1.5 (University of Kiel, Germany).

## 3. Results and Discussion

**Figure 2:**
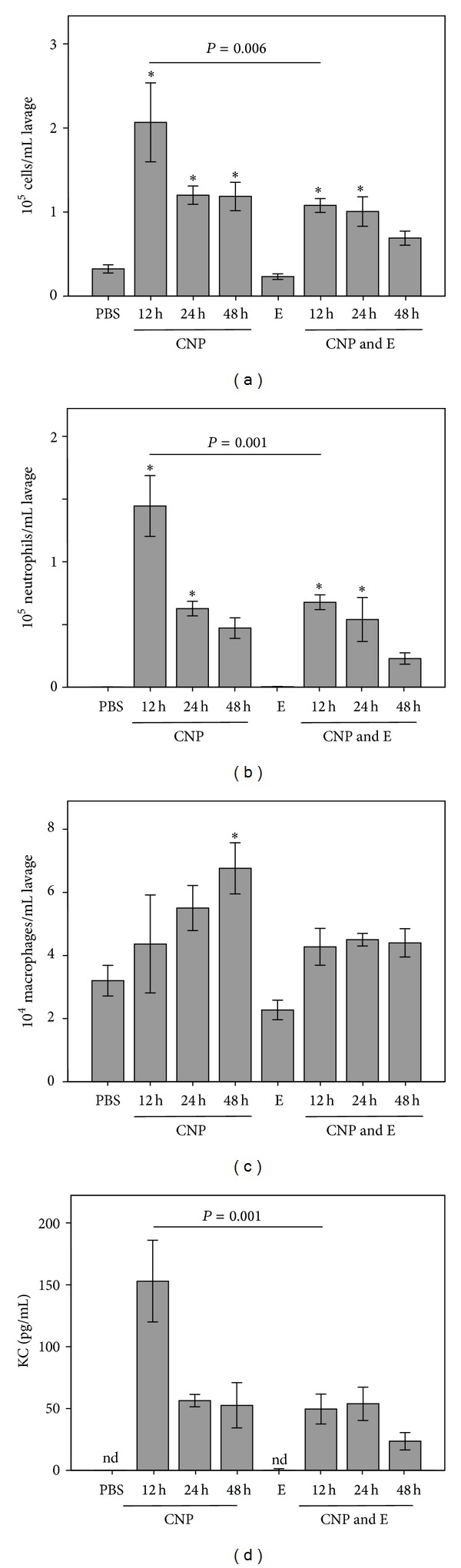
Time course of lung inflammation after single application of 5 mg/kg CNP in the presence or absence of ectoine (1 mM). Animals (*n* = 5) were analysed after the indicated time points. (a) Total number of cells per mL BAL; (b) total number of neutrophilic granulocytes per mL BAL; (c) total number of macrophages and monocytes per mL BAL; (d) pg/mL of KC in BAL. *, significantly different to the respective control (PBS or ectoine) after Tukey's HSD post hoc testing considering multiple testing; E, ectoine; nd, not detectable.

With the current studies we aimed at investigating the effect of ectoine in an in vivo experimental system suitable as a model for allergic airway diseases. In a first approach, lung inflammation in C57/Bl6 mice was investigated with respect to dose response after 24 h (Figure 1). Analyses of bronchoalveolar lavage (BAL) demonstrate a dose dependent increase of inflammatory cells. The neutrophil influx is reflected by the increase of the neutrophil recruiting cytokine KC. The effect of ectoine was then tested in a time course experiment in which animals were exposed for 12 h, 24 h, and 48 h with 5 mg/kg CNP in the presence or absence of ectoine (Figure 2). Control animals were exposed with saline (PBS) or ectoine solution for 24 h. After a maximum of total cell counts as well as neutrophil numbers in BAL after 12 h, the inflammatory parameters attenuated during the observation period. The reduction of neutrophils was counteracted by the increase of macrophages, which help to clear the lung from apoptotic neutrophils. In the presence of 1 mM ectoine, the kinetics of the neutrophilic inflammation were mirrored at a lower level, indicating the preventive potential of the substance in the mouse system with the most striking statistical significance after 12 h.

**Figure 3:**
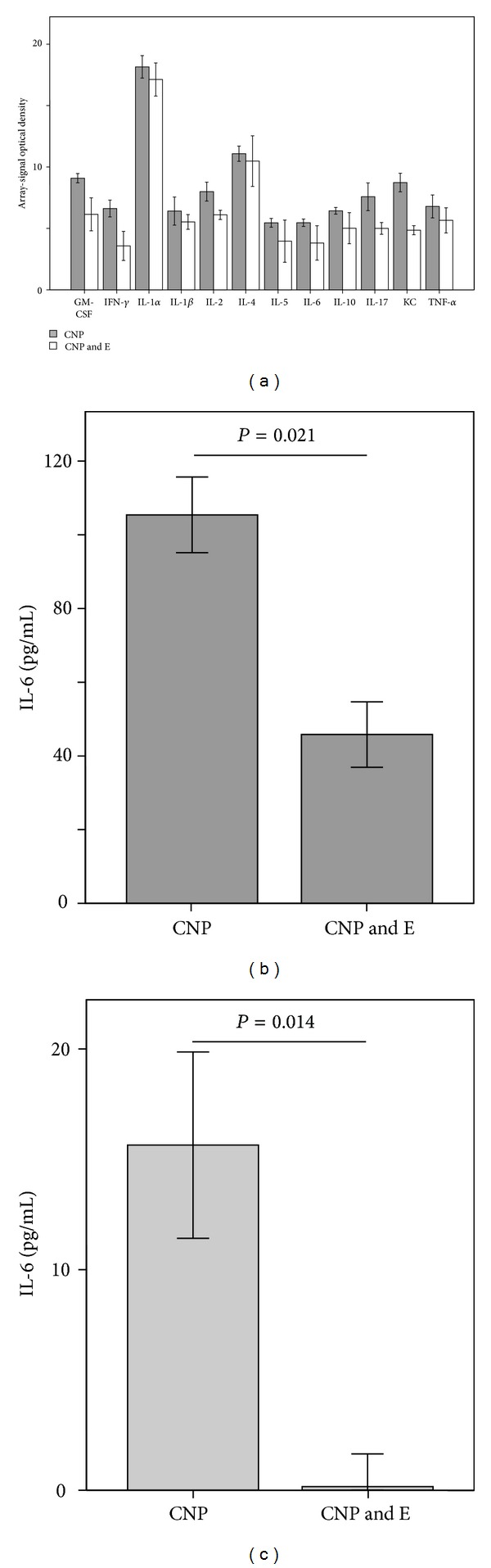
Effect of ectoine treatment on inflammatory mediators 12 h after exposure. (a) BAL analyses of two animals from each group. Given error bars are based on duplicates from both measurements. Significant values cannot be calculated. (b) IL-6 in BAL of 5 animals; (c) IL-6 in serum of 5 animals. CNP, 5 mg/kg carbon nanoparticles; E, 1 mM ectoine. Significant values (two sided) in (b) and (c) were calculated by Mann-Whitney *U*-test.

The consequences of this reaction were furthermore tested at the level of cytokines and chemokines using membrane arrays for two BAL samples from each 12 h exposure group (Figure 3(a)). Although the differences in cytokine patterns are based on a low number of individuals, the general reduction of the selected inflammatory markers by the ectoine is obvious. In addition to these analyses, the potential of ectoine to reduce systemic inflammation was investigated by measuring IL-6 in BAL and in serum of exposed animals (Figures 3(b) and 3(c)). IL-6 levels in control animals were not detectable. The application of ectoine together with CNP led to a significant reduction of cytokine levels both in BAL and in serum of the animals. In particular, the reduction of IL-6 in the serum of the animals treated with ectoine in addition to CNP highlights the potential of this kind of treatment to reduce systemic effects of the lung inflammation. IL-6 has been discussed as a serum marker for asthma which also might be involved in the pathogenesis of asthma [15]. This result may be an indication that ectoine has also beneficial effects with respect to the allergic sensitization which might be boosted by environmental pollution.

**Figure 4:**
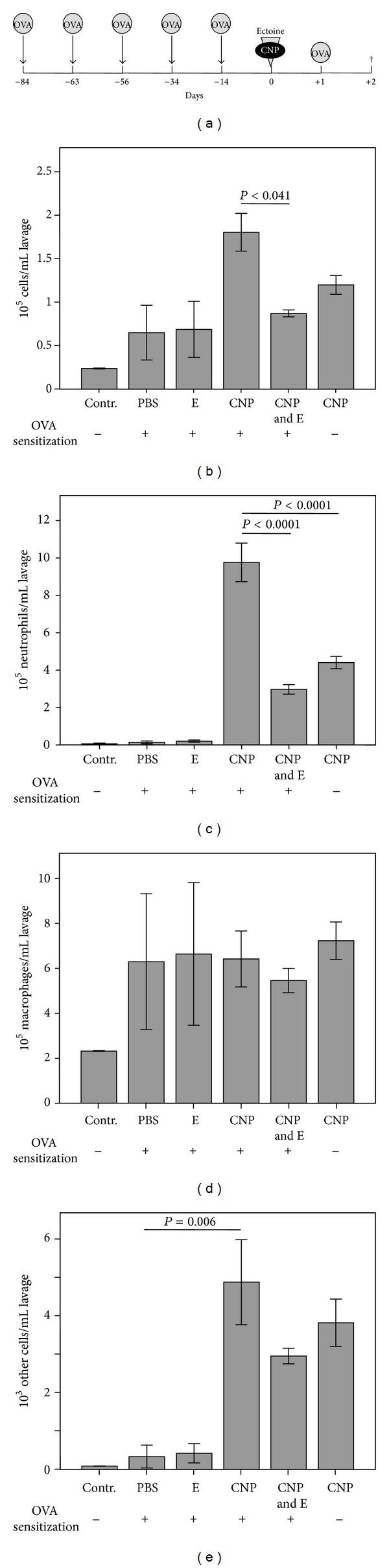
Effect of CNP and ectoine on OVA provocation-induced lung inflammation in sensitized animals (control groups *n* = 3 and exposure groups *n* = 5). (a) Experimental design. Animals were sensitized by repetitive intraperitoneal OVA application. At day 0, 5 mg/kg CNP in the presence or absence of 1 mM ectoine was applied to the animals. OVA provocation by inhalation (1%, 30 min) was performed 12 h after treatment. Measurements were made 24 h after challenge. (b) Total number of cells per mL BAL; (c) total number of neutrophilic granulocytes per mL BAL; (d) total number of macrophages and monocytes per mL BAL; (e) total number of other cells including lymphocytes and eosinophilic granulocytes.

The potential of ectoine to reduce the exacerbation of the immune reaction by the neutrophilic lung inflammation was then tested in animals which were sensitized for 12 weeks by repetitive injection of the model allergen ovalbumin (OVA). Animals were challenged 12 h after the application of particles in the presence or absence of ectoine (Figure 4(a)). Inflammatory parameters in the lung of the animals were investigated 24 h after the provocation. At this time point, all sensitized animals exhibited elevated cell numbers in BAL which were highest in CNP-exposed animals (Figure 4(b)). This reaction proved to be significantly reduced when ectoine was applied together with the particles. The differential analysis of the cells revealed that the exacerbating effect of the particles as well as the preventive effect is mostly due to the changes in neutrophilic granulocytes. Remarkably, the same effects are observed at the level of lymphocytes and eosinophils which are considered relevant for the allergic response (Figure 4(e)). Due to the very low number of these cells and the overwhelming number of neutrophils, these cell types were analysed together (other cells). Although exhibiting high heterogeneity, macrophage numbers were elevated in all challenged animals irrespective of an existing inflammation during the provocation. As observed in earlier experiments, this effect was not influenced by the ectoine treatment (Figure 4(d)).

Considering that ectoine itself has no antioxidant capacity the current data may give an indication for the mechanisms by which environmental particles contribute to asthma exacerbation. In this scenario, ectoine significantly reduces the neutrophilic inflammation induced by the particles at the time point of the onset of the antigen provocation. Ectoine is known not to interact with the particles and it does not scavenge reactive oxygen species. It is therefore plausible that the exacerbating effect of combustion-derived nanoparticles is mediated by the proinflammatory trigger which can be observed at the level of neutrophil influx and the respective proinflammatory mediators.

Due to the molecular mechanisms by which ectoine acts, the substance in a first line was suggested to be used as a preventive agent against environmental particulate air pollution. Ectoine in epithelial cells has been shown to prevent typical effects of cell stress triggered by combustion-derived nanoparticles [16]. This strategy however was not designed to replace attempts to improve ambient air quality but was rather considered for predisposed persons who may suffer from chronic lung inflammation or from allergic diseases of the airways. It was therefore important to test whether in the situation of an immune response which is exacerbated by the presence of environmental model particles the inflammatory outcome can be reduced by ectoine. The data show significant differences in neutrophil numbers 36 h after the challenge, indicating that the number of neutrophils recruited by the immune response on OVA provocation can be reduced. At this time point eosinophilic infiltration has just started. Other studies observe elevated levels of these cell types later after the challenge [17]. Therefore the analysis of other cell types in our system has to deal with very low absolute numbers. Although statistically not significant, a trend in the reduction of these cell types by ectoine can also be observed. In order to investigate the effect of ectoine on this particular immune reaction, long-term experiments have to performed. It has to be tested whether one single ectoine application is sufficient to reduce the accumulation of eosinophils in the lung or whether repetitive treatments are necessary.

In humans different immunological phenotypes of asthma are described. Besides asthma which is characterised by eosinophilic granulocytes, neutrophilic asthma as probably environmentally induced disease is observed [18]. Like COPD, this disease is dominated by a stable chronic neutrophilic lung inflammation which often is not sensitive to the treatment with glucocorticoids [19]. Together with our earlier studies, which demonstrate an effect of ectoine on neutrophils in vivo and ex vivo [14], the current data suggest that ectoine might also be efficient in the treatment of neutrophilic asthma and should be tested in this context.

## 4. Conclusions

From the data presented here, we conclude that ectoine has beneficial effects on the exacerbation of airway immune responses by environmental particulate air pollution. After having revealed deeper insight into the value and mechanisms of ectoine in chronic neutrophilic lung inflammation, the recent data can be considered the first approach to apply this preventive strategy to predisposed persons like asthmatics. Furthermore, the study indicates the mechanistic importance of neutrophilic lung inflammation in the exacerbation of allergic airway diseases.
